# Jerzy Jurka – 1950–2014

**DOI:** 10.1186/s13100-014-0031-3

**Published:** 2015-01-09

**Authors:** Prescott Deininger

**Affiliations:** Department of Epidemiology, Tulane Cancer Center, Tulane University School of Public Health and Tropical Medicine, New Orleans, LA 70112 USA

The passing of Dr. Jerzy Jurka (Jurek to his friends) represents a heartfelt loss to his many friends in the Mobile DNA field. The whole field of Mobile DNA has lost the scientific efforts of a brilliant colleague and for many of us an esteemed friend. The accompanying biography provides an outstanding outline of his life and career, so I will focus primarily on his impact to the field of Mobile DNA.

Jurek’s first publication related to Mobile DNA was the discovery of an Alu element in an alpha globin gene in the late 1980s while working with Temple Smith. Jurek was one of the pioneers of well-trained bioinformatics experts to focus his expertise on mobile elements, with one of the early and important findings of subfamilies in the Alu elements. Through the years, his ability to handle large genomic datasets led him to a number of important discoveries, including a bioinformatic prediction of the target site for SINEs and LINEs, the discovery of several new families and clades of mobile elements, as well as overall distribution, evolution and structure of mobile elements in whole genomes. Among his more interesting new mobile elements were the discovery of helitrons and polintrons, identified from whole genome sequence analyses.

In the process of his analyses, Jurek developed several new bioinformatic tools, including MASE, an early program for multiple alignment editing useful for aligning repetitive sequences, and CENSOR, a program for identifying various classes of repetitive DNA sequences in genomic sequences. However, I believe that his real strength in this research was his ability to couple his understanding of bioinformatics with a genuine understanding of biological processes. This led him to make some amazing findings in terms of genome evolution, evolution of mobile elements, and even functional conclusions such as the likelihood of an endonuclease sequence preference leading to the preferred Alu insertion sites. These studies resulted in over 120 publications, making many significant and even groundbreaking discoveries in the field of mobile elements.

As outlined in the accompanying biography, Jurek’s career did not always take a traditional track. After training in several laboratories in Europe, he went to the University of Michigan for postdoctoral training. However, it was as a fellow with Temple Smith at the Dana Farber Cancer Institute that he began his love affair with mobile elements. He then briefly joined BIONET in the San Francisco Bay area which was associated with the early days of GENBANK. He was greatly influenced at that time by his interactions with two great minds who originally proposed the molecular clock model that was so critical to Jurek’s future work, Emile Zuckerkandl and Linus Pauling. Jurek served as the Assistant Director of Research for the Linus Pauling Institute for several years.

In 1994, Jurek founded the Genetic Information Research Institute (GIRI) in the Bay Area and served as its president until his death. Jurek often stated that the strong support of his wife, Elzbieta, was critical to his ability to work through an independent research institute. Through the Institute, he supported his research on mobile elements and mentored many other outstanding young bioinformatics scientists. However, the Institute also served as a tremendous resource to our entire Mobile DNA community, largely funded by grants from the National Library of Medicine, to database information on mobile elements in a database called REPBASE. This database, and its associated online publication REPBASE Updates, represented the ultimate collection of mobile element sequence data for members of the Mobile DNA community. Critically, this database serves as the reference data for sequence analysis programs like REPEATMASKER to identify mobile elements in genomes.

To further facilitate development of our field, over the last decade, Jurek organized and held three outstanding meetings on the “Genomic Impact of Eukaryotic Transposable Elements.” These meetings brought a wide variety of scientists together to discuss the structure, evolution and function of mobile DNA, including workshops for those wanting to utilize bioinformatics tools more effectively. These extremely influential meetings are often referred to as the “Asilomar” meetings by investigators in the field.

Dr. Jerzy ‘Jurek’ Jurka was not only an outstanding scientist, he was a tremendous leader for our field. He was an investigator who loved a passionate scientific discussion, and equally loved to discuss politics and the family of which he was so proud. He has left an indelible impression on our field with his seminal contributions. He will be greatly missed.

